# Supplementary material for: Yiai Fuzheng decoction inhibits triple-negative breast cancer by remodeling the immune microenvironment
Source: Front Immunol. 2025 Sep 30;16:1615631. doi: 10.3389/fimmu.2025.1615631 (PMC12518410; doi:10.3389/fimmu.2025.1615631)
Supplement: Supplementary file 2 [file DataSheet1.zip › Supplementary File 2/Treatment/D_vs_H/02_PCA/All_Groups/All_Combinaiton/PC23_Score_2D_Label.pdf]

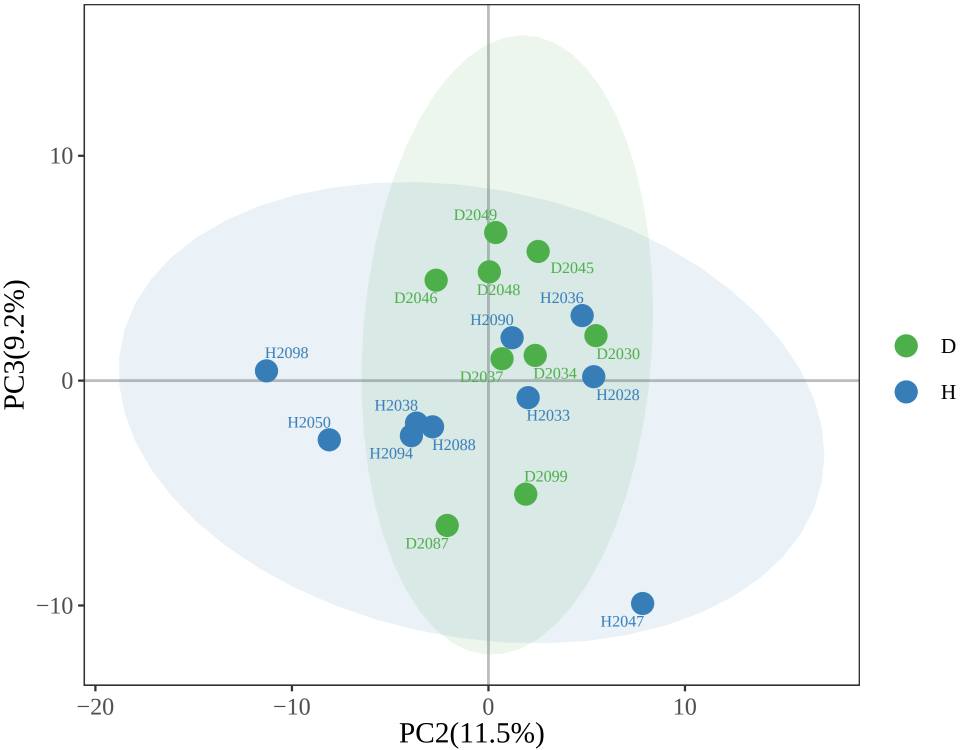

PC3(9.2%)
PC2(11.5%)
10
0
−10
−20
−10
0
10
D2049
D2045
D2048
D2046
H2036
H2090
D2030
H2098
D2034
D2037
H2028
H2033
H2038
H2050
H2088
H2094
D2099
D2087
H2047
D
H
